# From Prevention to Disease Perturbations: A Multi-Omic Assessment of Exercise and Myocardial Infarctions

**DOI:** 10.3390/biom11010040

**Published:** 2020-12-30

**Authors:** Melanie T. Odenkirk, Kelly G. Stratton, Lisa M. Bramer, Bobbie-Jo M. Webb-Robertson, Kent J. Bloodsworth, Matthew E. Monroe, Kristin E. Burnum-Johnson, Erin S. Baker

**Affiliations:** 1 Department of Chemistry, North Carolina State University, Raleigh, NC 27606, USA; mtodenki@ncsu.edu; 2 National Security Division, Pacific Northwest National Laboratory, Richland, WA 99354, USA; kelly.stratton@pnnl.gov (K.G.S.); lisa.bramer@pnnl.gov (L.M.B.); 3 Biological Sciences Division, Pacific Northwest National Laboratory, Richland, WA 99354, USA; bobbie-jo.webb-robertson@pnnl.gov (B.-J.M.W.-R.); kent.bloodsworth@pnnl.gov (K.J.B.); matthew.monroe@pnnl.gov (M.E.M.); 4 Department of Biostatistics and Informatics, University of Colorado, Aurora, CO 80045, USA; 5 Environmental Molecular Sciences Laboratory, Pacific Northwest National Laboratory, Richland, WA 99354, USA; kristin.burnum-johnson@pnnl.gov; 6 Comparative Medicine Institute, North Carolina State University, Raleigh, NC 27695, USA

**Keywords:** lipidomics, metabolomics, multi-omics, planned myocardial infarction (PMI), myocardial infarction (MI), exercise, heart, cheminformatics

## Abstract

While a molecular assessment of the perturbations and injury arising from diseases is essential in their diagnosis and treatment, understanding changes due to preventative strategies is also imperative. Currently, complex diseases such as cardiovascular disease (CVD), the leading cause of death worldwide, suffer from a limited understanding of how the molecular mechanisms taking place following preventive measures (e.g., exercise) differ from changes occurring due to the injuries caused from the disease (e.g., myocardial infarction (MI)). Therefore, this manuscript assesses lipidomic changes before and one hour after exercise treadmill testing (ETT) and before and one hour after a planned myocardial infarction (PMI) in two separate patient cohorts. Strikingly, unique lipidomic perturbations were observed for these events, as could be expected from their vastly different stresses on the body. The lipidomic results were then combined with previously published metabolomic characterizations of the same patients. This integration provides complementary insights into the exercise and PMI events, thereby giving a more holistic understanding of the molecular changes associated with each.

## 1. Introduction

For decades, physical activity and diet have been considered the primary preventative strategies for numerous diseases, including cardiovascular disease (CVD). As the leading cause of death worldwide, rigorous characterization of CVD and the subsequent incidences of myocardial infarction (MI) are crucial for reducing its occurrence [1]. Despite the prevalence of CVD and resulting MI events worldwide, the complex pathophysiology underlying CVD origins has yet to be fully defined [2]. Even with advancements such as diagnosis with CK-MB and cTn assays and methods for CVD prediction from traditional risk factors alone or in tandem with molecular predictors, CVD-related events continue to be the leading cause of death worldwide [1,3,4,5]. Thus, improving our understanding of these disease mechanisms could serve to reduce the current morbidity rate of CVD by providing more effective prevention, intervention and treatment strategies.

In CVD and other diseases, such as type 2 diabetes, osteoporosis and some forms of cancer, there is a well-recognized, negative correlation with the intensity, duration and continuation of exercise events [6,7,8]. Since exercise subjects the heart to hemodynamic stress and overloading of pressure and volume [8], morphological adaptation of the heart occurs following recurrent exposure to exercise, effectively diminishing the risk of heart disease by reducing cholesterol and suppressing hypertension and atherogenesis [9,10]. However, over-exertion of the heart muscle from exercise can result in calcification that limits the capacity of the heart to pump blood, thereby increasing the risk of cardiovascular events [11]. On the other hand, a sedentary lifestyle along with high blood pressure, abnormal blood lipid profiles, smoking and obesity are all major risk factors for CVD, typically triggering the development of an intermediate phenotype prior to a MI [12,13,14]. Therefore, elucidating a balance between the beneficial and detrimental mechanisms of exercise is crucial for optimizing heart performance and reducing CVD risk and mortality rates [13].

While exercise and diet can be preventative, certain people are genetically predisposed to CVD and other heart diseases. Therefore, leveraging models of stroke and MI events responsible for 80% of CVD end-stage phenotypes provides additional molecular information about treatments and the induced injuries. Hypertrophic cardiomyopathy (HCM) is the most prevalent heritable cardiac disease, estimated to be present in 1 out of every 500 individuals [15,16]. Obstructive HCM (HOCM) is a subtype mechanistically defined by the barricaded outflow of the left ventricular heart cavity at rest (1/3 of cases) or at provocation (1/3 of cases) [15,16]. The reduction of left ventricular outflow in HOCM cases culminates in increased left ventricular pressure, high wall stress, impaired left ventricular filling, myocardial ischemia and a reduced cardiac output [16,17]. Currently, aspirin, β-blockers and pacemakers are all common remediation strategies to mitigate these symptoms [16,17]. Failure of these therapeutic approaches to alleviate left ventricular blockage, however, requires removal of obstructing tissues through either surgical excision or alcohol septal ablation (ASA), where an injection of alcohol triggers a planned myocardial infarction (PMI) and reduces the left ventricle blockage caused by systolic anterior motion of the mitral valve [16]. While both procedures have had similar patient outcomes and survival rates, ASA treatment and the resulting PMI have proven to be a less invasive approach preferable for surgically at-risk patients [16,17]. Evaluating the molecular changes occurring from a PMI also grants researchers tremendous insight into the pathophysiology of spontaneous MI events that plague one American every 40 s with a global mortality rate of CVD-related events accounting for 31% of the deaths in 2015 [18,19].

Mass spectrometry (MS) has become a popular analytical tool to characterize molecules changing in biological systems through omic studies. While the annotation of a singular “ome” (i.e., proteome, lipidome, metabolome) elucidates significant aspects of disease pathophysiology, comprehensively characterizing a disease through one class of biomolecules does not provide the holistic information often needed. Thus, multi-omic measurements, wherein multiple classes of biomolecules are analyzed and integrated, provide a greater understanding of molecular interplay and pathophysiology [20]. For example, since metabolites and lipids both reflect immediate changes occurring in a system, together they allow for an investigation into early-stage perturbations [21,22]. Furthermore, lipids have routinely been linked to exercise and MI mechanisms [2,23,24,25], so their combination with metabolites provides a complementary way to assess system dysregulation. In this study, lipidomic assessments were performed on plasma taken from two cohorts; the first cohort’s samples were taken before and one hour after exercise performed with a specific treadmill testing procedure, and the second cohort’s samples were taken before and one hour after a PMI. The lipidomic results were then compared to a targeted polar metabolite study of the same patient cohorts [26,27], and together, the multi-omic comparison provided a more comprehensive characterization of the various biomolecule classes altered upon different stressors of the body and heart. This comparison therefore allowed for the exploration of molecular differences between CVD-related events and preventative strategies within humans.

## 2. Materials and Methods

### 2.1. Sample Extraction and Data Collection

#### 2.1.1. Human Sample Collection and Extraction

**Figure 1:**
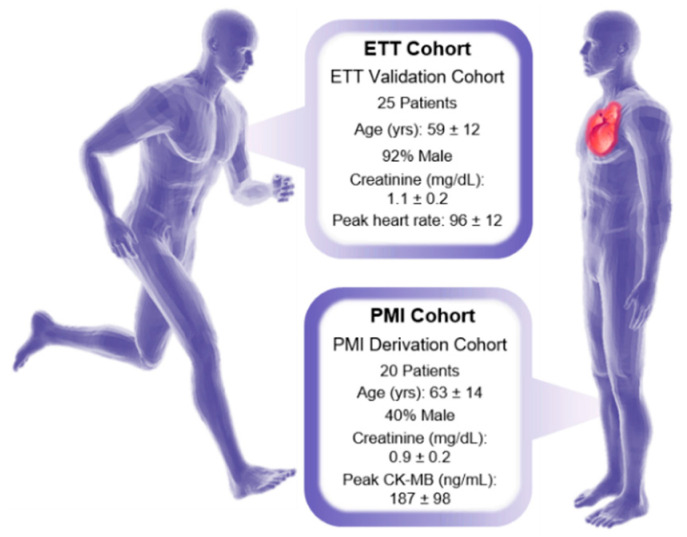
Demographics for the PMI cohort (**left**) and ETT cohort (**right**). Continuous variables are given as mean ± standard deviation and categorical variables are shown as percentages.

Both an exercise and a PMI cohort were evaluated in this manuscript, and informed consent was obtained from all human participants in the studies. In the exercise cohort, plasma samples were collected from the periphery veins of 25 patients before and one hour following exercise treadmill testing (ETT) [26]. In the PMI cohort, plasma samples were also collected from the periphery veins of an additional 20 patients before and one hour following a PMI [27]. The paired before and after sampling of the same patient for both studies was performed to yield a high statistical power despite the limited number of samples analyzed, since the before sample could be used as the control for each patient [28]. An overview of the patient demographics for both the ETT and PMI cohorts is given in Figure 1 and Supplemental Table S1. Additional cohort information is also expanded upon in the original publications [26,27]. Notably, the male demographic of the exercise cohort was large compared to the PMI study, wherein women were in the majority [26]. Additionally, in the exercise study, enrolled patients had to meet a normal exercise tolerance criteria, which included having an estimated peak VO_2_ capacity over 70%, a heart response rate exceeding 85% predicted baseline and a pre-exercise fasting period of 4 h [26]. PMI patients were also monitored with CK-MB and troponin T assays, with peak levels observed at standard spontaneous MI times with CK-MB at 4.5 h and troponin T at 8 h following a PMI event [27,29]. The PMI derivation cohort were all primary HOCM cases with septal thickness ≥16 mm; resting outflow tract gradient ≥30 mmHg; inducible outflow tract gradient ≥50 mmHg; failed medical intervention; and appropriate coronary anatomy [27]. Targeted analysis of 210 metabolites was completed in the original publications for each cohort with a triple quadrupole mass spectrometer (AB4000Q; Applied Biosystem/Sciex, Farmingham, MA, USA), and detailed protocols on those methods can be found for the ETT study in Lewis 2010 [26] and PMI study in Lewis 2008 [27].

#### 2.1.2. Lipid Extraction

For the lipidomic study, lipids were extracted in 2 mL Sorenson tubes from 25 µL aliquots of plasma following a modified Folch protocol [30,31]. Briefly, 600 µL of a 2:1 mixture of −20 °C chloroform/methanol was introduced to the plasma samples which was then vortexed for 30 s. A phase separation was induced by adding 150 µL aliquots of HPLC grade water and then vortexed again for an additional 30 s. The samples then rested for 5 min at room temperature prior to centrifugation at 12,000 rpm for 10 min at 4 °C. Samples were then placed on ice where 350 µL aliquots of the bottom organic layer were removed, dried in a speedvac and then re-suspended in 250 µL of 2:1 chloroform/methanol for storage at −20 °C. Immediately before instrumental analysis, the total lipid extracts were dried down and reconstituted in 5 µL chloroform and 100 µL methanol. Pooled case and control samples for the exercise and PMI studies were generated by combining 5 µL aliquots of each before plasma sample separately.

#### 2.1.3. Lipidomic Instrumental Analysis

Lipidomic instrumental analysis of the 45 before and 45 after extracted human plasma samples was completed with an Agilent 6560 IM-QTOF MS platform (Santa Clara, CA, USA) outfitted with the commercial gas kit (Alternate Gas Kit, Agilent, Santa Clara, CA, USA) and a precision flow controller (640B, MKS Instruments, Andover, MA, USA). The LC–IMS–CID–MS data were collected in both positive and negative ESI from 50–1700 *m*/*z* with a 1 sec/spectra cycle time. Reverse phase liquid chromatography (RPLC) separation was completed with a 10 μL sample injection onto a Waters CSH column (3.0 mm × 150 mm × 1.7 µm particle size) on a Waters Acquity UPLC H class system (Waters Corporation, Milford, MA, USA). Separation of lipid species was achieved with a 34-min LC gradient (mobile phase A: acetonitrile/water (40:60) containing 10 mM ammonium acetate; mobile phase B: acetonitrile/isopropyl alcohol (10:90) containing 10 mM ammonium acetate) at a flow rate of 250 μL/min as described in Table 1. A 4-min column wash and 4-min equilibration were also used as described in Table 2.

### 2.2. Data Processing

#### 2.2.1. Lipid Identification

Accurate mass tag (AMT) matching within LIQUID software was used to assign all lipid identifications [32]. The LC–IMS–CID–MS platform typically allows for the assignment of head group and fatty acyl (FA) structural moieties of each uniquely identified lipid species using the criterion of mass accuracy below 5 ppm, precursor and fragment peak alignment across dimensions, and CCS values < 2% different from the reference value. While head group annotation is largely unambiguous, FA assignment is more complex due to the propensity of isomers. From the collision induced dissociation (CID) measurements, the number of carbons and double bonds is generally achieved; however, additional specifics, such as *sn*-backbone position, double bond position or double bond orientation, are often indistinguishable in these studies [33]. Therefore, the most confident lipid speciation achieved through this analysis included the head group and individual fatty acyl groups with unknown *sn*-positions, as denoted by “_” (i.e., PC (16:0_18:2)) [34]. For lipids where individual FA constituents could not be identified, the summed carbon and double bond counts are noted, e.g., PC (34:2). Any features matching more than one lipid identification are separated by a “;” to denote both as potential matches. Furthermore, isomeric experimental observations were assigned “_A”; “_B”; etc., to denote the observed chromatographic and/or IMS separation of these species. The peak areas of the 352 lipids identified in the exercise study (262 from positive mode, 85 in negative mode and 5 in both modes) and the 299 lipids identified in the PMI study (225 in positive, 72 in negative and 2 in both modes) were exported as a “.csv” format for processing and statistical assessment regarding each before/after paired comparison (Supplemental Tables S2 and S3).

#### 2.2.2. Data Processing and Statistics

Statistical analysis of the targeted polar metabolites was carried out as detailed previously [26,27]. Briefly, in the targeted annotation of 210 metabolites in the ETT and PMI studies, 20 were found to be statistically significant one hour following exercise (16 upregulated and 4 downregulated) and 13 were statistically significant one hour following a PMI (7 upregulated and 6 downregulated) at a Benjamini–Hochberg corrected *p* ≤ 0.005 cut-off. Processing and statistics of the lipidomics data also followed the same procedures, where statistical significance was determined from log_2_ transformed abundances using MetaboAnalyst (version 4.0, Edmonton, AB, CA) [35]. The ETT statistical analysis was completed using a paired t-test, and the PMI comparisons were completed with a Wilcoxon signed-rank paired t-test due to their unequal variance. A Benjamini–Hochberg multiple comparison correction was also applied for both analyses with a significance cut-off of *p* ≤ 0.005 to match the previously published metabolite statistics [36]. Interestingly, no statistically significant lipids were observed one hour following ETT, whereas the PMI study yielded 207 statistically significant lipids: 66 upregulated and 141 downregulated (Supplemental Tables S2 and S3). Comparison of sex in the PMI cohort and ischemia in the ETT cohort was completed to account for additional differentiation following the above protocols for each cohort. No significant species were detected from either comparison.

### 2.3. Data Interpretation

#### 2.3.1. Lipidomics Data Interpretation

Lipidomic relationships were investigated using cheminformatics to interrogate structure-function associations across head groups and fatty acyl (FA) moieties [37,38,39]. Head group clustering was completed with the SCOPE toolbox [39]. Here, SMILES [40] obtained from LipidMaps [34] for each lipid identification were clustered by structural similarity using an ECFP_6 fingerprint [41], Tanimoto distance and complete linkage using the *fingerprint* and *ggtree* packages in R (Version 3.6.2, Vienna, Austria) [42,43]. Lipids with multiple LipidMaps matches were cataloged by a representative SMILES for hierarchical clustering. To facilitate the visualization of head-group trends, pigmentation of dendrogram nodes was used to denote lipid classes. FA tail presence was further assessed by selectively parsing out lipids by FA composition. For our analyses, most *sn*-1 and *sn*-2 fatty acyl positions were unknown, so all possible positions were considered to account for potential *sn*-positional effects. Lipids with multiple identities were partitioned into all possible identifications to visualize each potential FA contribution to significance. Summary statistics (adjusted *p*-value, log_2_ fold change) of lipids were subsequently overlaid with the *pheatmap* package in R [42,44]. Color gradients of red (upregulated) and blue (downregulated) were applied to visualize significance with darker colors indicating a larger fold change (log_2_FC) or smaller *p*-value (adjusted *p*-value), while grey values represented identified but not statistically significant lipids.

#### 2.3.2. Multi-omics Data Interpretation

Hierarchical clustering was again utilized to assess the multi-omic association of statistically significant metabolites and lipids. Dendrograms provided visualization of the structurally similar and statistically significant species (BH adjusted *p*-value ≤ 0.005), both individually and in tandem. Metabolite clustering was accomplished with MAACS keys fingerprint, Tanimoto distance and complete linkage using *fingerprint* and *ggtree* packages in R (Version 3.6.2, Vienna, Austria) from each SMILES representation [42,43]. The resulting metabolite dendrograms allowed for a summary of the significant species following exercise and PMI events where adjusted *p*-values followed the same gradient as described above. Node color in the metabolite dendrogram was used to annotate the biological roles attributed to each metabolite. Conversely, in the multi-omics dendrogram built using ECFP_6 fingerprint, Tanimoto distance and complete linkage, all metabolites were grouped together in a single node color because of the relatively small number of statistically significant metabolites relative to lipids.

## 3. Results

The previous targeted metabolomic study for both the ETT and PMI cohorts provided great insight into statistically significant polar metabolites [26,27,45], but overlooked important nonpolar molecules changing due to each event. The recent annotation of lipids in both CVD and exercise has elucidated the critical roles these molecules serve in each event [24,46,47,48,49,50,51,52,53,54,55,56]. Therefore, the inclusion of lipidomic and multi-omic assessments in this manuscript provides a more in-depth profile of ETT and PMI molecular mechanisms.

### 3.1. Lipid Identifications and Statistical Significance

**Figure 2:**
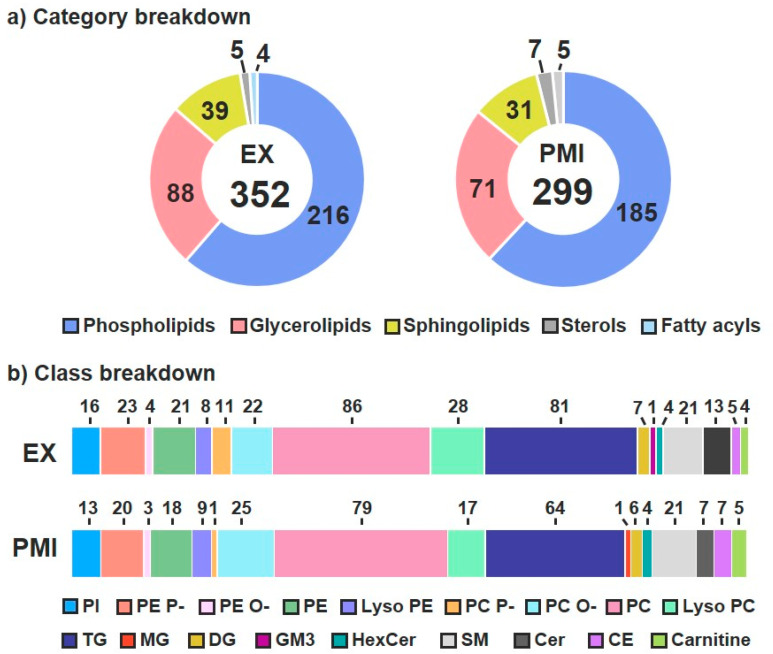
Identified lipid category and class coverage. (**a**) Five lipid categories were observed for plasma from patients in both the ETT (**left**) and PMI (**right**) cohorts. (**b**) In the class breakdown, the majority of the lipids fall within the sphingolipid, glycerolipid and phospholipid categories.

To perform both the ETT and PMI lipidomic analyses, multi-dimensional assessments were carried out by leveraging a LC–IMS–CID–MS instrumental platform [32,38]. The LC–IMS–CID–MS analyses yielded a total of 352 unique lipid identifications for the ETT cohort and 299 for the PMI cohort across the same five lipid categories: glycerolipids, sphingolipids, phospholipids, fatty acids and sterols [57]. The 352 ETT lipids were composed of 216 phospholipids, 88 glycerolipids, 39 sphingolipids, 5 sterols and 4 fatty acids (Figure 2a, left); the PMI cohort had 185 phospholipids, 71 glycerolipids, 31 sphingolipids, 7 sterols and 5 fatty acids (Figure 2a, right). The breakdown of lipid category designation into classes showed both studies having: three phospholipids (phosphatidylinositols (PIs), phosphatidylcholines (PCs) and phosphatidylethanolamines (PEs)), three sphingolipids (sphingomyelins (SM), ceramides (Cer) and hexose ceramides (HexCer)), two glycerolipids (triacylglycerolipids (TGs) and diacylglycerolipids (DGs)), one sterol (cholesteryl ester (CE)) and one FA (carnitine) (Figure 2b). Additional diversity within the phospholipids was observed in the FA linkages (including alkenyl ether (plasmalogen; P-) and alkyl ether (O-)) and FA numbers (e.g., lyso and diacyl species). Only a few lipid species were specific to each cohort including a ganglioside (GM3) belonging to the sphingolipid category in the ETT cohort and a monoacylglycerol (MG) from the glycerolipid category observed in the PMI cohort.

**Figure 3:**
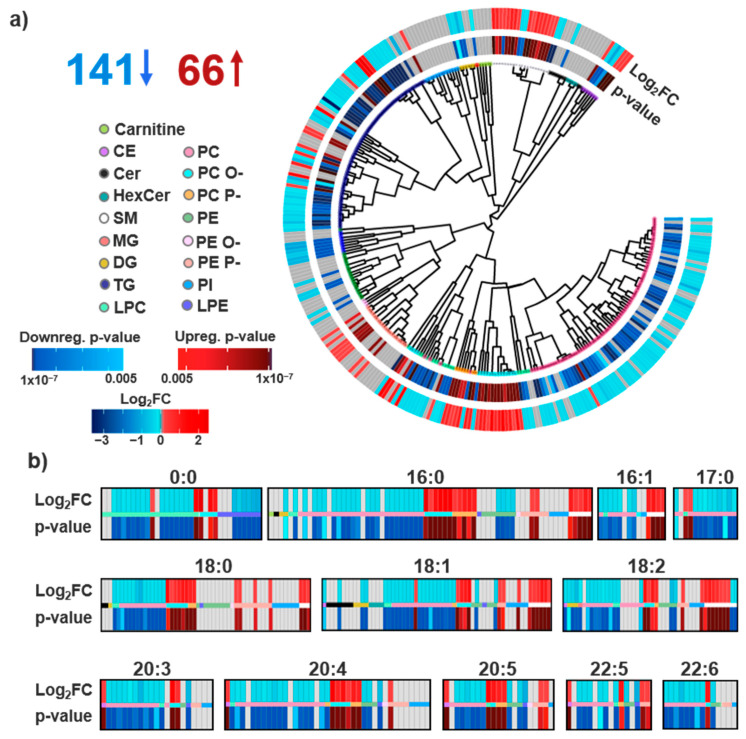
Lipids detected and statistically significant in the PMI comparison. (**a**) Of the 299 uniquely identified lipids, 141 were statistically downregulated and 66 were upregulated following a PMI with a *p*-value cut-off of 0.005. The lipid head group associations are visualized in a circular dendrogram with *p*-values (inner ring) and Log2FC (outer ring) statistics overlaid simultaneously for each lipid identification. (**b**) FA lipid composition was also investigated by plotting all unique FA components. Statistically significant lipids are shown in red and blue for up- and downregulation, and identified lipids lacking statistical significance are shown in grey. The magnitude of variation for Log_2_FC and adjusted *p*-values are visualized through a color gradient, with darker colors indicating a more significant *p*-value or larger fold change.

Of the identified lipids, a drastic difference was observed in statistical significance for the exercise and PMI cohorts. One hour after ETT, no lipids were found to be statistically significant, even after further assessment of metadata, including gender, age and BMI. However, we do note our statistical criteria were very stringent to compare them with the previous metabolomics studies, so directly above our significance cutoff we observed lipids of interest within the lyso PC, GM3, PE P-, DG and carnitine classes. Specifically, we noted the largest fold changes for PC (20:5_0:0), carnitine (10:1) and carnitine (14:1), which had values of −1.28, −1.29 and −1.18 FC. The lipidome changes in PMI, however, told a completely different story. An hour after a PMI, 207 lipids (69% identified) were statistically significant, even with the stringent criteria, with 141 downregulated and 66 upregulated (Figure 3a). To further evaluate the PMI lipids, we utilized head group and FA composition to visualize structure–function relationships of the statistically significant species. Head group associations of all identified lipids were clustered by their structural similarity [34,40,42]. The resulting circular dendrogram is shown in Figure 3a with the adjusted *p*-value in the inner ring and log_2_FC on the outer ring. The most consistent observation relating to head groups was the upregulation of PC O-, PC P- and PE P-. The upregulation of SM lipids, another component of lipid bilayers abundantly present in lipid rafts and integral in cholesterol homeostasis, was also observed in the PMI study [58]. Conversely, PC lipids which have overlapping roles as charged species enriched within the outer lipid membrane layer were downregulated following a PMI [59]. Additionally, a general downregulation of glycerolipids was also detected following a PMI event, a contradictory finding to the positive correlation of TGs and MI incidence [60,61] This finding may instead reflect FAs serving as the primary energy substrates of the heart where non-esterified FAs, products of glycerolipids degradation, are rapidly complexed with CoA [62]. Notably, ceramides which have previously been positively correlated with cardiac disease risk were not observed to be statistically significant in our PMI cohort [63]. Exceptions to the head group trends, however, were noted for almost every class discussed herein. For example, we observed split dysregulation across SMs, CEs and other classes, illustrating effects beyond just head group influence.

Discrepancies between lipid head group composition and biological dysregulation suggest additional selectivity likely attributable to the FA components of lipid structures. Within FAs, important differences include chain length, and double bond number, position and orientation [64]. Previous efforts have elucidated FA chain length to directly influence cardiac pathology, but plasma studies have been less successful in capturing this effect [24]. To explore these associations, we further interrogated FA dysregulation in the identified lipids, as shown in Figure 3b. While the findings in these plots mainly correlated with the head group analyses, a few FA-specific observations could be extracted. First, a FA dependence of CE differential expression was observed—with 18 carbon-containing CEs being downregulated and CEs with 20 and 22 carbon PUFAs being upregulated. Long chain polyunsaturated fatty acids (LC-PUFAs) are a class of FAs characterized as having 18 or more carbons and at least two double bonds, often serving as precursors to lipid mediators [65]. PUFA dysregulation was also recognized among PE, PC and PE P- lipids; PE and PC lipids containing PUFA tails were downregulated, while the majority of significant PE P- lipids were upregulated. In an additional assessment of the summed FA double bond number, it was observed that while the majority of glycerolipids were statistically downregulated, the upregulated TG species had a greater number of unsaturation sites compared to the downregulated species. This is in agreement with models for predicting CVD onset, which have included unsaturated TG species [4,24]. However individual FA information was not attained for the majority of the TG species due to difficulties in assigning their MS/MS spectra.

### 3.2. Multi-Omics Results

**Figure 4:**
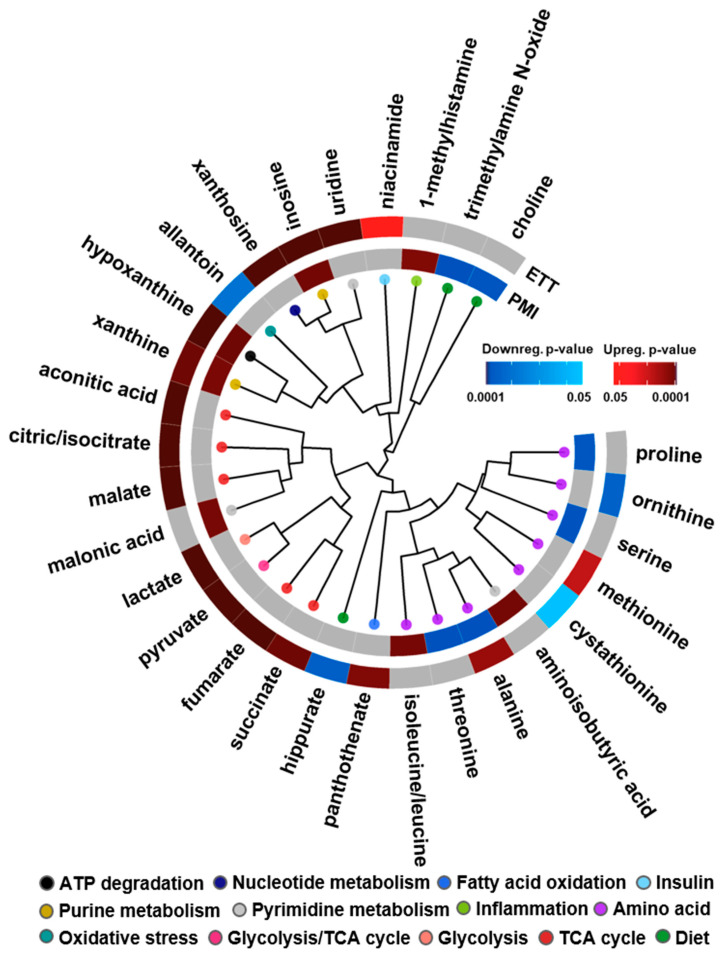
Statistically significant metabolites in the ETT and PMI studies. A circular dendrogram is utilized to showcase the differentially expressed metabolites in the PMI (inner ring) and ETT (outer ring) cohorts. Red and blue are used for up- and downregulation with a color gradient visualizing the magnitude of the adjusted *p*-value observed. Grey metabolites were detected but not statistically significant.

To assess how polar and nonpolar molecules change in both the ETT and PMI cohorts, the lipidomic results were integrated with the previously performed targeted analysis of 210 polar metabolites [26,27]. Results from these analyses elucidated both unique and shared statistically significant metabolites and biological processes across both events (Figure 4). For example, glycolysis and TCA cycle metabolites (red, pink and peach nodes) were upregulated following ETT, a finding agreeable with the known mechanisms of burning energy through high-intensity exercise [66]. Additionally, niacinamide, a component of NADH that is also associated with energy through its interaction with insulin, was found to be statistically upregulated with exercise. In PMI, the dietary metabolites of PC lipids previously shown to predict CVD risk, choline and trimethylamine N-oxide (TMAO), were downregulated and clustered next to each other to affirm their structural relationship [18]. Amino acid dysregulation was also observed following both ETT and PMI, as alanine was statistically significant through its upregulation immediately following exercise but downregulation following a PMI. Both ETT and PMI also shared an upregulation of xanthine and hypoxanthine, metabolites involved in purine metabolism and ATP degradation, which are notably upregulated following cellular damage. These xanthine metabolites can also interact with xanthine oxidase to produce reactive oxygen species, a mechanism well characterized in heart failure [67].

**Figure 5:**
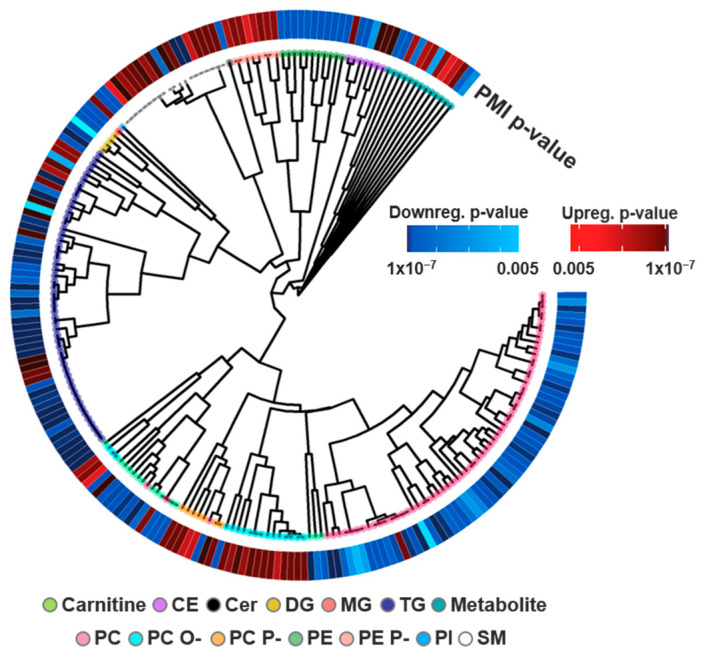
The multi-omic assessment of statistically significant lipids and metabolites from a PMI event. Adjusted *p*-values for each molecule are shown around the dendrogram. Red and blue are used for up- and downregulation with a color gradient to visualize magnitude.

From our analysis comparing the lipidomic changes in ETT and PMI, we note unique profiles where plasma metabolite signals best characterized mechanistic changes following high-intensity exercise training. Conversely, we demonstrated an overwhelming dysregulation of lipids following a PMI in the end-stage phenotype of CVD, in addition to the metabolomic findings that were previously published. While the metabolomic analyses elucidated changes for both the ETT and PMI cohorts with slight overlap between each characterization, the lipidomics results were quite different. While no statistically significant lipids were noted in the ETT study, the sheer number of statistically significant lipid associations in the PMI cohort (207, 69% of identified lipids) provide striking evidence for the integral role of the lipidome immediately following a PMI event (Figure 5). The findings from the ETT cohort were, however, in accordance with other exercise studies which have observed lipidome disruption being proportional to the duration and intensity of exercise [68]. Previous characterizations of lipid variation in exercise have centered on the decrease in free carnitine and increase in short-chain acylcarnitine through its crucial capability of shuttling FAs into mitochondria within muscle tissue for energy usage [25,69]. Dysregulation of carnitines has faced some disagreement in literature, likely from the lack of correlation between muscle and plasma sampling [69]. Further, the energy sources of exercise differ substantially as low intensity training relies on fat as a primary fuel source, while high intensity training uses carbohydrates as an immediate energy supply [23]. From the observation of metabolite intermediates of glycolysis and the TCA cycle such as lactate being upregulated, we feel we can confidently state that known mechanisms of high intensity training were taking place in our cohort [26]. From the dysregulation of energy processes in the metabolomics data and the variation in carnitine species observed just above the significance cutoff, it is possible that these species were in fact perturbed in our system as has been noted by others (Supplemental Table S2) [52,69]. A variety of factors may preclude this annotation, including age-based impairment of the acyl carnitine pathway that diminishes FA oxidation and study-to-study variation from different exercise training regimes [54,70]. A lack of differential expression of the lipidome following exercise may also reflect that lipid variation is not always immediate [24]. The singular treadmill training event for this analysis, therefore, may be too short to assess any additional lipidomic changes [52]. From the known pathophysiology of over-exercise triggering the calcification of the heart muscle, the activation of lipid enzymes by Ca^2+^ may suggest more drastic lipidome dysregulation would be observed with repeated exercise training [24].

From our analysis of 20 patients before and after a PMI event, we observed several instances of lipid dysregulation with substantial biological implications. Ether lipids (PC O-, PC P-, PE P-) have been shown to be disproportionately abundant in brain and heart tissues as components of the lipid bilayer with unknown biological implications [71]. While the biological significance of ether lipids overexpressed in heart tissue is not fully understood, the upregulation of these species in plasma following a PMI is likely indicative of tissue degradation following ASA treatment. However, only a subset of membrane lipids were upregulated, suggesting these lipid classes carry additional significance for ASA-induced PMI. This finding could potentially be explained by the preferential oxidation of O- and P- linkage sites that serve to protect the *sn*-2 FA group from oxidation [72]. In the heart, dysregulation of PC lipids in tandem with increased activity of phospholipase enzymes has been observed in CVD, where lysophospholipids contribute to atherosclerosis and vascular damage through their role in inflammation, as was observed here in their downregulation [73,74]. Altogether, the obstruction of heart tissue following a PMI could serve dual purposes, reflecting both a breakdown of ablated cellular tissue and dysregulation of essential biological processes, such as energy production and inflammation. PUFAs with a double bond on the third carbon (n-3) have previously been shown to serve preventative roles in CVD through their antiatherogenic effects and may explain the dysregulation of PUFA-containing lipids [75]. Metabolomic analysis of the PMI samples showed the most dysregulation among amino acids, where branched amino acids are precursors for glutamine and alanine synthesis in muscles [76]. Conversely, amino acids associated with cardiac remodeling (proline) were downregulated [77].

### 3.3. Study Comparison

**Figure 6:**
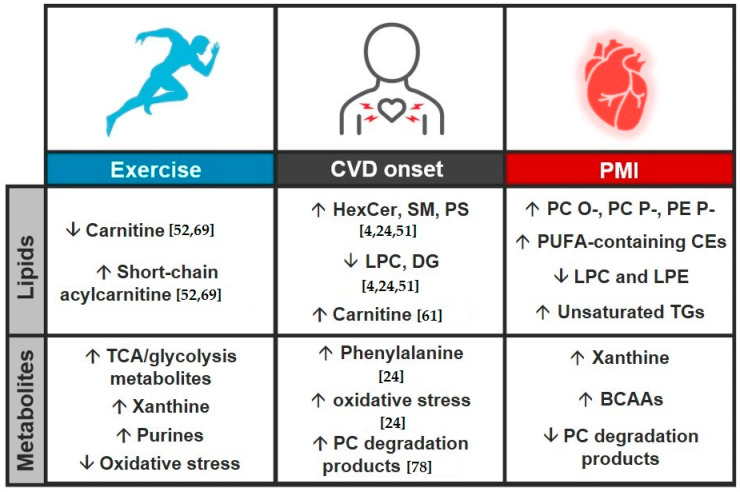
Comparison of lipidomic and metabolomic trends for exercise (**left**), cardiovascular disease (CVD) onset (**middle**) and planned myocardial infarction (PMI) model (**right**). Results include a summary of observed results from this and the referenced previous studies noted by citation number in the figure [4,24,51,52,61,69,78].

Combining the two complementary stories of metabolite and lipid dysregulation before and after exercise and a PMI provides an important assessment of their biological changes (Figure 3 and Figure 4). This comparison is incredibly insightful for understanding CVD pathophysiology, as shown by comparing our results with the general consensus of molecular dysregulation from several exercise, CVD onset and MI studies (Figure 6) [4,24,51,52,61,69,78]. Results from the CVD onset studies have illustrated upregulation of sphingolipids and carnitines, and shown downregulation of lyso PC and DGs. CVD onset metabolomic studies have also elucidated distinct molecular changes to include mechanisms of oxidative stress and PC degradation products promoting atherogenesis [55]. In the comparison of exercise, CVD onset and a PMI, there is quite substantial overlap in the perturbed processes, but the molecules being dysregulated are often unique. For example, different energy processes were dysregulated in both exercise and PMI, as glycerolipids were largely downregulated in PMI and TCA/glycolysis metabolites were upregulated with exercise. Differential expression of both 1-methyl histamine and lysophospholipids was also observed, and since both have been linked to roles in inflammation, this suggests a possible response to ASA treatment [73,74]. Uniquely, ether lipids, which were upregulated in PMI, are also recognized regulators of ion channels [71]. Relative to CVD onset, we noted a number of lipid and metabolite species dysregulated in both the ETT and PMI studies (Figure 6). For example, lysoPCs (LPC) were downregulated across PMI and are largely corroborated by literature [4,56]. The further annotation of choline and TMAO degradation products of PC lipids suggests an even greater significance in PC lipids for the development of end-stage perturbations, however the direction of change between CVD and the PMI model differed [55]. We also noted opposite trends when comparing CVD onset results from the literature and our ETT cohort; carnitines have been reported to be downregulated in exercise but upregulated in CVD onset, further reflecting the importance of the shift in energy processes between PMI and exercise [69,78]. These findings are significant for further elucidating the mechanisms of CVD, which we and others have shown reflect drastic changes in the lipidome but are missed from polar metabolomics experiments [4,48,51,53]. We would, however, like to note that the limited size of our patient study fails to capture sex-based differentiation of CVD onset established previously [79,80,81,82]. We also note limitations in our ETT analysis from a singular bout of exercise and disparities among patients from variables such as cardiovascular health history and ischemia that may hinder the elucidation of exercise-based lipid dysregulation.

## 4. Conclusions

The metabolomic and lipidomic findings observed for the exercise and PMI cohorts showcased their unique pathophysiology. Of the statistically significant metabolites observed for both events, little overlap was found, implicating unique molecular processes for each [26,27]. Since the insights from a singular class of biomolecules are inherently limited, we expanded the metabolomic analyses to include lipids. Novel instrumentation platforms and cheminformatics tools were applied to provide confident lipid identifications and investigate lipid variation [38]. The lipidomic analyses illustrated how the exercise cohort had no statistically significant lipids after treadmill testing, while 69% of identified lipids were dysregulated one hour after a PMI. This finding was in itself very interesting and distinguished the molecular mechanisms for the two events. As such, the polar metabolites were more informative for the exercise study, while the lipidomic results provided a better assessment of the PMI cohort. Specifically, one hour following a PMI, lipid species with head groups including PC O-, PC P- and PE P- were all upregulated, while SMs were mainly upregulated and PCs were mostly downregulated. PUFAs were also selectively dysregulated across lipid head groups following a PMI. However, even with the lipid structural insight achieved, discrepancies in class trends were still observed, since LC–IMS–CID–MS analyses allow for the confident assignment of lipids, but analytical improvements are necessary to probe the roles of double bond position and orientation in these discrepancies. Interestingly, integrating the multi-omic exercise and PMI studies showed perturbation of energy processes across both events. The multi-omic analyses also corroborated findings from singular omic analyses where inflammation and atherogenic processes are heavily implicated in PMI. Furthermore, their comparison with CVD onset studies showed strong agreement between the lipid and metabolite dysregulation observed in the PMI cohort, and less agreement with the ETT cohort results, as expected. Ultimately, the integration of the lipid and metabolite data elucidated unique biological roles within molecular classes, providing complementary profiles for how preventative strategies and MI events greatly differ in their molecular mechanisms.

## Figures and Tables

**Table 1:** Lipid elution gradient.

Time	% MPA	% MPB	Flow Rate (mL/min)
0	60	40	0.25
2	50	50	0.25
3	40	60	0.25
12	30	70	0.25
15	25	75	0.25
17	22	78	0.25
19	15	85	0.25
22	8	92	0.25
25	1	99	0.25
34	1	99	0.25

**Table 2:** Lipid column wash.

Time	% MPA	% MPB	Flow Rate (mL/min)
34.5	60	40	0.3
35	1	99	0.3
35.5	1	99	0.3
36	60	40	0.35
37	60	40	0.3
38	60	40	0.25

## Data Availability

Raw data is available through MassIVE (https://massive.ucsd.edu/MSV000086620).R Code for recreating data visualization within this manuscript is available at https://github.com/BakerLabNCSU/PMI_Exercise_Multiomics.

## Supplementary Materials

The following are available online at https://www.mdpi.com/2218-273X/11/1/40/s1, Table S1: ETT and PMI cohort information. Table S2: Exercise results. Table S3: PMI results.
